# Prenatal Syphilis Screening Mandates and Maternal Syphilis Case Detection

**DOI:** 10.1001/jamahealthforum.2026.0123

**Published:** 2026-03-20

**Authors:** Sarah E. Baum, Leila Agha, Nicolas A. Menzies, Jessica Cohen

**Affiliations:** 1 Department of Global Health and Population, Harvard T. H. Chan School of Public Health, Boston, Massachusetts; 2 Department of Health Care Policy, Harvard Medical School, Boston, Massachusetts

## Abstract

**Importance:**

Rates of congenital syphilis in the US have surged over the past decade, despite most states having long-standing mandates requiring clinicians to offer syphilis screening early in pregnancy. Gaps in screening coverage remain, and first-trimester screening alone may miss cases. Several professional bodies now recommend repeat screening in the third trimester and at delivery. Evidence on the impact of expanded prenatal syphilis screening mandates on case detection is limited.

**Objective:**

To evaluate the effectiveness of expanding prenatal syphilis screening mandates on syphilis case detection during pregnancy.

**Design, Setting, and Participants:**

Birth certificate data from 33 US states between 2012 and 2022 were analyzed using a staggered difference-in-differences design. Maternal syphilis case detection in 4 states that enacted mandates for third-trimester and delivery screening (Arizona, Georgia, Louisiana, and Michigan) were compared with 29 control states without such mandates during this period. The new mandates required all pregnant people be offered third-trimester screening. Three of the states further required that individuals at high risk of syphilis infection be offered screening again at delivery, and 1 state required universal delivery screening. To ascertain whether expanded mandates were associated with changes in screening coverage, inpatient discharge records from 1 mandate expansion state (Georgia) were analyzed. Data were analyzed from December 2024 to September 2025.

**Exposures:**

Passage of a universal syphilis screening mandate in the third trimester and a high-risk or universal mandate at delivery between 2012 and 2022.

**Main Outcomes and Measures:**

Maternal syphilis case detection (cases per 100 000 live births) and the share of deliveries receiving syphilis screening.

**Results:**

The study sample included 16.3 million live births and 20 961 reported syphilis cases between 2012 and 2022 in 4 mandate expansion states and 29 control states. Expanded screening mandates were associated with a 26% (95% CI, 3-53) increase in maternal syphilis case detection in the first quarter after enactment. The increase in case detection attenuated thereafter and was no longer significant within 1 year (11%; 95% CI, −17 to 48; *P* = .48).

**Conclusions and Relevance:**

In this study, expanded prenatal syphilis screening mandates may improve syphilis case detection in the near-term but are unlikely to have sustained impact without complementary efforts, such as those that facilitate clinician adherence and ensure patient access to and completion of treatment.

## Key Points

**Question:**

What is the association between mandates requiring prenatal syphilis screening in the third trimester and delivery and maternal syphilis case detection?

**Findings:**

In this cross-sectional study including 16.3 million live births and 20 961 reported syphilis cases between 2012 and 2022 in the US, maternal syphilis case detection increased by a statistically significant 26% in the first quarter after mandates at the third trimester and delivery were enacted. The magnitude of this effect declined within the following year, although it remained positive.

**Meaning:**

Mandates for syphilis screening later in pregnancy may briefly increase maternal syphilis case detection but are unlikely to generate sustained impact on their own without complementary supports for screening and treatment.

## Teaser

This cross-sectional study evaluates the effectiveness of expanding prenatal syphilis screening mandates on syphilis case detection during pregnancy.

## Introduction

Congenital syphilis rates in the US have surged nearly 12-fold over the last decade (from 8.7 to 105.8 cases per 100 000 live births between 2013 and 2023) since reaching historically low rates in the early 2000s.^1,2^ Transmitted from mother to child during pregnancy, congenital syphilis increases the risk of stillbirth, neonatal death, low birth weight, and infant death, and can confer lifelong medical complications.^3,4^ Stark inequities persist: compared with White individuals, congenital syphilis rates are nearly 12-fold higher among pregnant people who identify as American Indian or Alaska Native and 4-fold higher among Black individuals. Congenital syphilis rates are also 6-fold higher among Medicaid beneficiaries compared with those with private insurance.^5,6^ Rising syphilis incidence among reproductive-aged women has been attributed to multiple factors, including diversion of public health funding for sexually transmitted infections, closure of clinics that offer sexually transmitted infection screening, and changes in access to reproductive health care.^5,6,7^

Congenital syphilis is almost entirely preventable with routine prenatal screening and prompt treatment for detected cases prior to delivery.^4^ Since prenatal syphilis is often asymptomatic or presents with nonspecific symptoms, clinical detection requires diagnostic testing. As of 2022, more than one-third of birth parents of affected infants did not receive timely testing, even though most women had received some prenatal care.^1^ Screening coverage is highly variable across locations and patients and often does not meet existing state requirements or recommendations by major professional bodies.^8,9,10,11,12^ In 6 states with laws requiring syphilis screening at least once in pregnancy, screening coverage among Medicaid enrollees ranged from 56% to 91%.^12^

Although clinicians have been legally required to offer screening in the first trimester in many states since the 1970s, first-trimester screening alone may miss cases.^13^ Since 2008, several states have enacted legislation requiring that clinicians offer repeat screening later in pregnancy (ie, third trimester or delivery) and to different segments of the pregnant population—either requiring screening for all pregnant individuals (universal screening) or only individuals at high risk of syphilis infection or without evidence of prior screening (high-risk screening).^14^ Third-trimester screening (between 28 and 32 weeks) can improve detection and prevent transmission of infections that are acquired later in pregnancy, seroconvert after an initial negative test result, and among those who delayed prenatal care or were not screened despite seeking care.^15,16,17^ Screening at delivery ensures that infants born with congenital syphilis are identified and receive prompt treatment to clear infection and limit further complications.

Repeat syphilis screening is now often part of the standard clinical guidance for pregnancy care. Joint guidelines from the American College of Obstetricians and Gynecologists and the American Academy of Pediatrics, as well as from the US Centers for Disease Control recommended screening all pregnant people early in the first trimester.^18,19^ Since 2012, all 3 organizations have recommended screening individuals at high risk of infection in the third trimester and at delivery. In 2024, the American College of Obstetricians and Gynecologists issued updated recommendations for clinicians to screen all pregnant people 3 times in pregnancy.^20^ In 2025, the US Preventive Services Task Force reaffirmed recommendations for universal screening early in pregnancy.^21^

Several factors may shape how effective mandates are at improving maternal syphilis case detection. They may not increase screening coverage; clinicians may not change how often they offer screening or patients, who can decline mandated offers of screening, may not take it up. Even if coverage does increase, the bulk of screening may be targeted to low-risk individuals and thus have a negligible impact on case detection.

There is limited evidence on the effectiveness of syphilis screening on maternal case detection.^22^ Two observational studies found that increasing syphilis screening during pregnancy—through a provincewide free screening program in China and through opt-out screening in a hospital emergency department in Chicago, Illinois—increased case detection.^23,24^ However, these studies cannot directly speak to the effectiveness of enacting expanded screening mandates in the US on case detection. Additionally, while some studies have descriptively assessed whether updates to clinical recommendations or state mandates change screening for other infectious diseases in pregnancy, no studies have rigorously evaluated this in the context of syphilis. To address these gaps, we evaluated the association between mandates expanding prenatal syphilis screening and maternal syphilis case detection using national birth certificate data.

## Methods

### Data and Outcomes

We obtained data on all live births from the National Center for Health Statistics for all 50 states and the District of Columbia from January 2012 through December 2022. We restricted our analysis to all live births reported using the 2003 Revised Live Birth Certificate. States adopted this revision at different times prior to full coverage in 2016, resulting in an unbalanced repeated cross-section. This study was declared nonhuman subjects research by the Institutional Review Board of the Harvard T. H. Chan School of Public Health. We followed the Strengthening the Reporting of Observational Studies in Epidemiology (STROBE) reporting guideline.^25^

We aggregated quarterly counts of syphilis infection using an indicator for a confirmed diagnosis or documentation of treatment.^26^ The indicator for syphilis infection was assumed to reflect infection status during pregnancy and at delivery, consistent with the official guidelines for completing this section of the birth record, which advise the health care professional to consult the prenatal record, labor and delivery admission form, admission history, and delivery record.^26^ For deliveries without evidence of a positive syphilis diagnosis or treatment, it was not possible to distinguish whether these individuals were not screened or whether they had received a negative test result. Presence of syphilis infection was recorded as unknown in 0.39% of live births; in our analyses, we grouped these deliveries with unknown infection status together with deliveries reporting no evidence of infection. Information on the frequency and timing of when screening occurred was not recorded. To our knowledge, there are no existing validation studies comparing infections reported on the birth certificate to official health department surveillance reports.

The primary outcome was the state’s quarterly maternal syphilis rate per 100 000 live births, calculated as the number of recorded syphilis infections among women who delivered in a quarter (regardless of parity) divided by the total number of live births in that quarter. Although syphilis infection can contribute to stillbirth, we did not analyze stillbirth as an outcome in this study due to limited power; the screening rate among stillbirths is low and the resulting number of documented syphilis-associated stillbirths is small.^27,28^ Race and ethnicity data were based on self-report.

### Exposure

Exposure was defined as delivering in a state after a mandate was expanded requiring prenatal syphilis screening be offered in the third trimester and at delivery between 2012 and 2022. We studied expanded screening mandates in 4 states (hereafter, *mandate expansion states*) with at least 2 years of observations posttreatment: Arizona, Georgia, Louisiana, and Michigan. Arkansas also enacted a screening mandate during our study period but was excluded from this analysis given the insufficient number of pretreatment periods for which outcome data were reported. California was excluded given the insufficient number of posttreatment periods. All 4 treated states in our sample already had an existing first-trimester screening mandate in place and enacted a universal mandate to offer screening in the third trimester and a mandate at delivery. The mandate at delivery was universal in Arizona and limited to high-risk individuals in Georgia, Louisiana, and Michigan (eTable 1 in Supplement 1). Births in 16 states that had already enacted a screening mandate at the third trimester or delivery prior to 2012 (hereafter, *always treated*) were excluded (eTable 2 in Supplement 1). Where the state of residence and delivery differed, births with any potential exposure to an expanded mandate in always treated states were removed.

### Statistical Analysis

We used a difference-in-differences design to compare changes in prenatal syphilis case detection in the 4 states that enacted an expanded screening mandate at third trimester or delivery compared with the 29 states that did not enact an expanded mandate (hereafter, *control states*) between 2012 and 2022. Model specifications are provided in the eMethods in Supplement 1.

We used two-way fixed effects accounting for staggered adoption to estimate the average treatment on the treated (ATT). This design relied on a parallel-trends assumption: changes in syphilis case detection in mandate expansion states would have continued on the same trend as control states in the absence of enacting an expanded mandate. We used a Poisson regression to model case detection on the log scale since the parallel-trends assumption may not hold on the linear scale if initial transmission rates differ across states.^29^ An event study model was used to evaluate case detection 8 quarters prior and 4 quarters after the mandate was enacted to assess the validity of the parallel-trends assumption in the premandate period. Standard errors were clustered at the state level and used to calculate 95% CIs. We converted the ATT on the multiplicative scale to the incidence scale following the approach outlined in Feng and Bilinski,^29^ as outlined in eMethods in Supplement 1.

We checked the sensitivity of our findings to several alternative specifications. We controlled for lagged annual state-level syphilis incidence excluding prenatal syphilis cases as well as share of births among birthing parents younger than 24 years; Hispanic, non-Hispanic Black, and non-Hispanic White birthing parents; those who completed high school or completed college; and those receiving Medicaid. We used a placebo treatment date of 1 year prior to when each state’s mandate was actually enacted. We also restricted the sample to data from 2014 to 2022 for a balanced repeated cross-section. Between 2012 and 2022, several control states issued provider letters, health alerts, or screening guidelines recommending expanded screening without enacting a mandate. We ran 2 additional analyses, excluding any control state that had issued a provider letter, health alert, or revised screening guidelines recommending expanded screening (n = 16) or that had only issued a provider letter or health alert (n = 12).

Since birth certificates only report syphilis cases and not whether screening was performed, we complemented our main analysis with descriptive evidence on temporal changes in screening following a mandate in 1 treated state. In 2015, Georgia implemented a universal mandate at the third trimester and a high-risk mandate at delivery. Risk was based on either not having been tested in the third trimester or disclosure of activities posing risk for infection occurring since the third trimester.^30^ State-level inpatient discharge records from Georgia for 2011 to 2019 from the Agency for Healthcare Research and Quality Healthcare Cost and Utilization Project State Inpatient Database were used to identify syphilis screening at delivery.^31^ Following previous literature, we identified all hospitalizations for childbirth using *International Classification of Diseases, Ninth Revision *(*ICD*-*9*) and *ICD*-*10 *and Diagnosis-Related Group (DRG) codes.^32^ Among all deliveries, we identified whether an individual was screened for syphilis using *Current Procedural Terminology* and *ICD* codes (eTable 3 in Supplement 1).^12,33^

All statistical tests were 2-sided, and *P* values less than .05 were considered statistically significant. Analyses were conducted using R version 4.2.1 (The R Foundation). Data were analyzed from December 2024 to September 2025.

## Results

The study sample included 16.3 million live births and 20 961 reported syphilis cases between 2012 and 2022 in 4 mandate expansion states and 29 control states. A total of 252 065 pregnant individuals (1.6%) were American Indian or Alaska Native, 784 863 (4.8%) were Asian or Pacific Islander, 2 302 020 (14.2%) were Black, 2 320 078 (14.2%) were Hispanic, 10 098 612 (61.9%) were White, and 426 431 (2.6%) were multiracial or another race. In the baseline period (2012 quarter 1 to 2014 quarter 2), mean quarterly syphilis case detection was nearly 3-fold higher in mandate expansion states (151 vs 49 cases per 100 000 live births), as was case detection of chlamydia and gonorrhea (Table). Relative to control states, pregnant individuals in mandate expansion states were more likely to be younger (mean [SD] of 35% [3] vs 31% [6] of births occurred in the 24 years or younger age category), non-Hispanic Black (mean [SD] of 29% [10] vs 7% [9]), have less than a high school education (mean [SD] of 16% [3] vs 13% [3]), have up to a high school education (mean [SD] of 29% [3] vs 24% [4]), and have been enrolled in Medicaid (mean [SD] of 52% [9] vs 39% [10]) and the Special Supplemental Nutrition Program for Women, Infants, and Children (mean [SD] of 50% [4] vs 39% [8]). They were less likely to be non-Hispanic White (mean [SD] of 56% [10] vs 71% [14]) and have completed college (mean [SD] of 33% [3] vs 40% [7]). Timing of prenatal care initiation was similar across mandate expansion and control states, with a mean (SD) of 75% (2) and 76% (5), respectively, initiating prenatal care in the first trimester.

**Table.** Characteristics of Deliveries in Baseline Period by State-Quarter (2012 Quarter 1 to 2014 Quarter 2)

Characteristic	Mean quarterly share of birthing parents, mean (SD), %		
	National (n = 375)	Mandate expansion states (n = 28)	Control states (n = 219)
Live births, No.	21 150	25 351	10 979
Incidence per 100 000 live births (cases, No.)			
Syphilis	62.0 (13.7)	150.7 (29.2)	48.8 (6.2)
Chlamydia	1910.9 (386.3)	2364.1 (558.7)	1843.4 (218.9)
Gonorrhea	260.1 (55.9)	407.4 (94.0)	217.3 (29.1)
Maternal age, y			
≤24	30.8 (5.7)	35.2 (3.4)	30.6 (6.2)
25-34	55.3 (3.6)	52.6 (2.0)	56.3 (4.0)
≥35	13.9 (3.8)	12.2 (2.0)	13.1 (3.4)
Race and ethnicity^a^			
American Indian or Alaska Native	2.1 (4.1)	0.5 (1.0)	3.4 (5.0)
Asian or Pacific Islander	4.4 (3.3)	3.2 (0.9)	3.8 (3.3)
Black	13.0 (11.7)	29.2 (9.5)	7.3 (9.1)
Hispanic	15.1 (12.5)	9.8 (6.8)	12.5 (10.9)
White	63.4 (16.6)	55.7 (9.6)	70.7 (14.3)
Other race	2.0 (1.8)	1.6 (0.6)	2.3 (2.2)
Timing of prenatal care initiation, mo			
1-3	74.5 (4.8)	74.6 (2.3)	75.6 (4.5)
4-6	19.6 (3.2)	19.1 (1.6)	19.1 (3.2)
>7 or None	5.9 (1.9)	6.3 (1.0)	5.2 (1.6)
Maternal educational attainment			
<High school	14.8 (3.6)	16.3 (2.5)	13.2 (3.3)
High school	24.4 (3.9)	28.6 (2.5)	23.7 (3.9)
Some college	22.1 (3.0)	22.6 (2.5)	22.9 (2.9)
≥College	38.7 (7.2)	32.5 (3.0)	40.2 (7.3)
Payer			
Medicaid	41.8 (9.7)	52.2 (8.9)	39.2 (10.2)
Private	48.9 (10.1)	40.6 (10.0)	50.8 (11.2)
Self-pay or other	9.3 (5.1)	7.3 (6.0)	10.0 (5.4)
Enrolled in WIC	41.6 (8.6)	49.6 (3.7)	39.0 (8.4)
Married	60.5 (7.2)	53.2 (4.5)	63.4 (7.7)
Immigrated	16.2 (8.7)	12.7 (4.9)	12.9 (6.3)

Abbreviation: WIC, Special Supplemental Nutrition Program for Women, Infants, and Children.

^a^ Race and ethnicity data were based on self-report. The other race category includes multiracial or other race.

**Figure 1.**
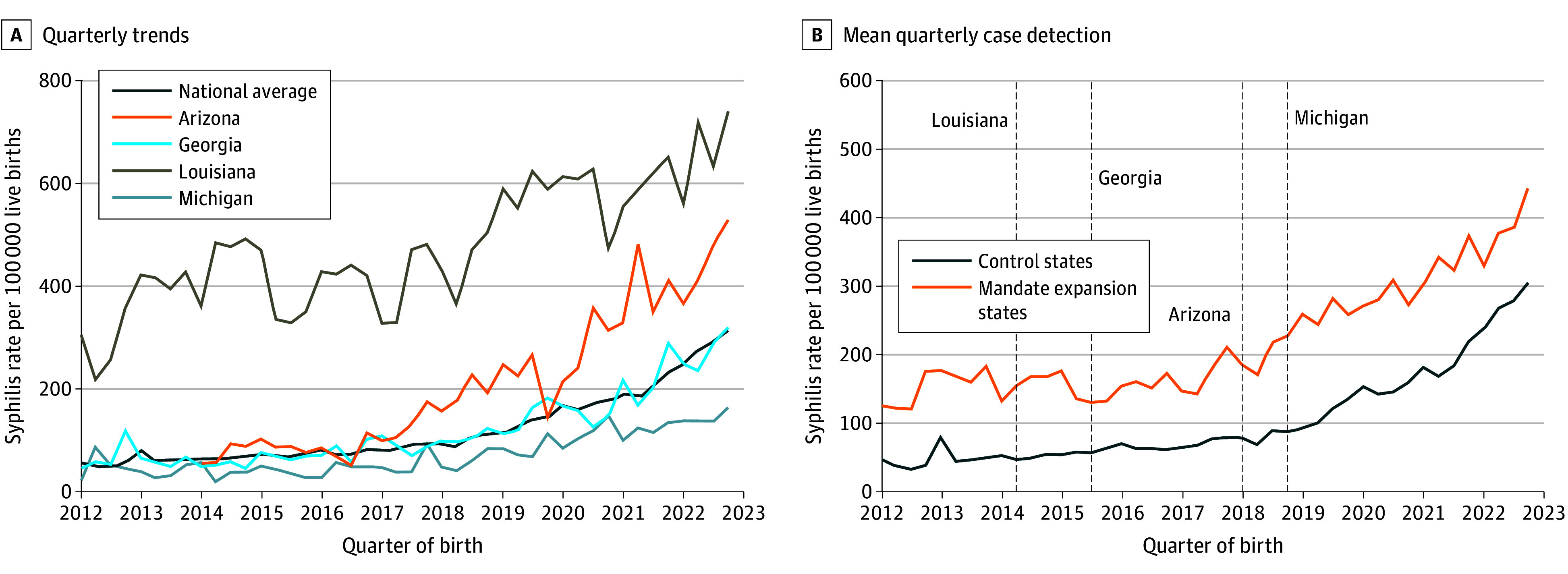
Line Graphs of Quarterly Trends in Maternal Syphilis Case Detection by State and Treatment Group From 2012 to 2022 Quarterly trends in maternal syphilis case detection per 100 000 live births from 2012 to 2022 from national birth certificates obtained through the National Center for Health Statistics. A, Quarterly trends in mean syphilis case detection nationally and for mandate expansion states. B, Mean quarterly case detection for the 4 mandate expansion states relative to 29 control states based on treatment group assignment (eTable 2 in Supplement 1). Dashed vertical lines indicate the quarter in which each mandate was passed in mandate expansion states (eTable 1 in Supplement 1). The US Centers for Disease Control and Prevention uses the same natality data to estimate the maternal syphilis rate. We confirmed that annual counts of maternal syphilis infection from the vital statistics data matched Centers for Disease Control and Prevention reports for 2016 to 2022, which is when they began reporting these data.^5^

Between 2012 and 2022, national maternal syphilis detection increased by 445% (from 58 to 316 cases per 100 000 live births) (Figure 1A). Case detection increased annually, with the greatest growth in 2019 (33% increase). In mandate expansion states, mean maternal syphilis detection increased by 252% (from 126 to 443 cases per 100 000) between 2012 and 2022 (Figure 1B). While their level of mean case detection was consistently lower relative to mandate expansion states, control states experienced a 524% increase between 2012 and 2022 (from 49 to 306 cases per 100 000 live births), with the greatest increase in growth occurring in 2022 (37.8% increase).

**Figure 2.**
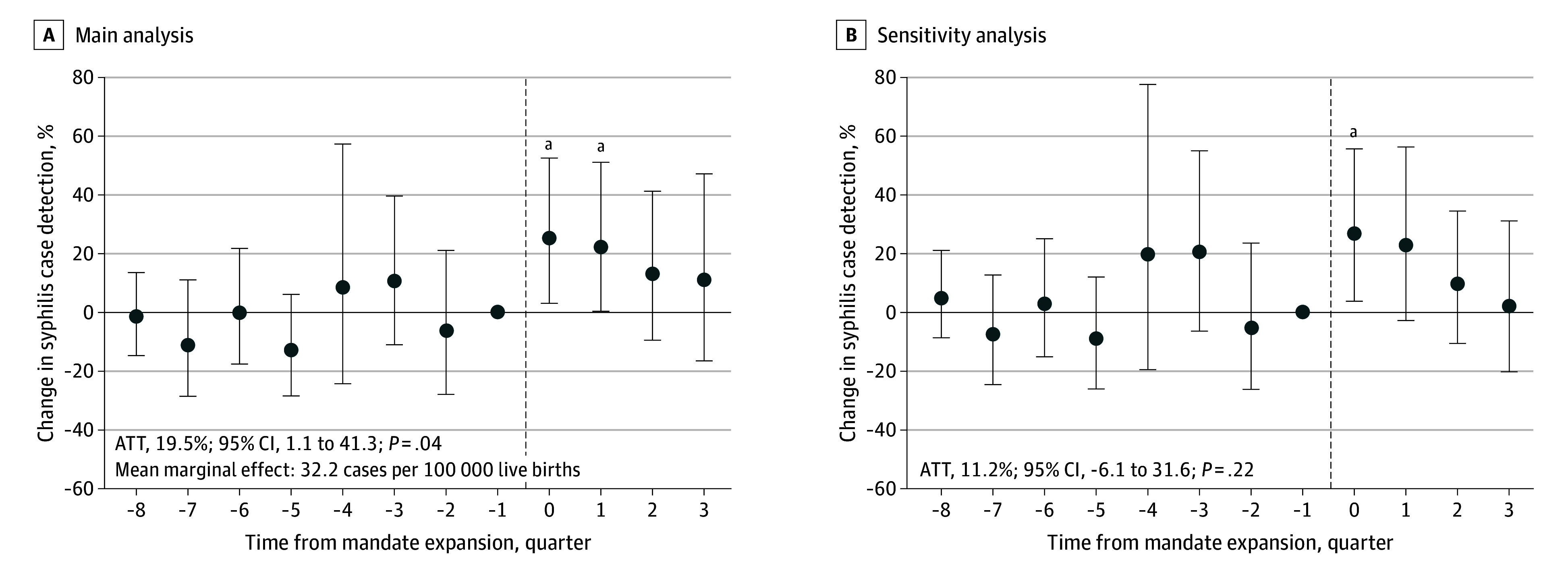
Dot Plots of Estimates of the Effect of Mandate Expansion on Quarterly Syphilis Case Detection Event study results from a staggered two-way fixed-effects specification (A) and from a specification controlling for lagged syphilis incidence in the population and time-varying demographic controls (eg, share of births among birthing parents younger than 24 years, with Hispanic ethnicity, non-Hispanic Black race, non-Hispanic White race, who completed high school, who completed college, and who received Medicaid) (B). Both specifications include state and quarter fixed effects. SEs are clustered at the state level with associated 95% CIs. Results are presented quarterly relative to the quarter preceding when each state legislature passed their screening mandate. The average treatment on the treated (ATT) represents the difference-in-differences estimates of the change in syphilis case detection per state-quarter was modeled using a two-way fixed-effects Poisson model for 4 quarters after the mandate was enacted. The mean marginal effect (or additional cases detected) was calculated using the approach described in Feng and Bilinski.^29^ The vertical dashed line indicates when the mandate was enacted. Error bars indicate 95% CIs. ^a^*P* < .05. ^b^*P* < .01.

Figure 2 presents event study results of maternal syphilis case detection 8 quarters prior to the passage of screening mandates and difference-in-difference results aggregated over 4 quarters after the mandate was passed. We did not find any differential trend in syphilis case detection in the preperiod and all 95% CIs included zero, suggestive of parallel trends. In the first quarter after the law was passed, incidence increased by 26% (95% CI, 3-53; *P* = .02) in mandate expansion states in our event study specification. This initial increase in detected syphilis cases dissipated over the subsequent 4 quarters, with no significant effect at 3 quarters after the mandate was enacted (11%; 95% CI, −17 to 48; *P* = .48). This trend was robust to the inclusion of controls; the magnitude of the initial increase was higher (28%; 95% CI, 4-56; *P* = .02) and continued into the second quarter (23%; 95% CI, −3 to 57; *P* = .08).

Overall, ATT estimates suggested that quarterly prenatal syphilis case detection was 19.5% (95% CI, 1-41; *P* = .04) higher than what it would have been if states had not enacted an expanded mandate. This translated into 32.2 additional detected cases per 100 000 live births per quarter after the mandate was enacted. ATT estimates varied when estimated separately for each mandate expansion state (eTable 4 in Supplement 1).

**Figure 3.**
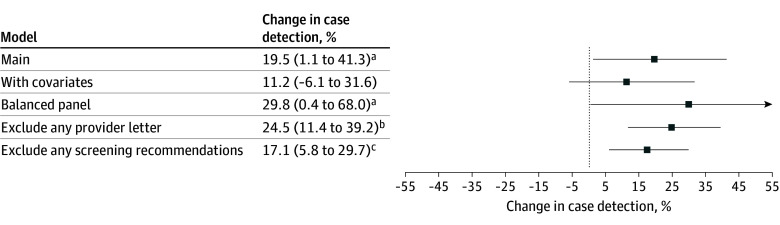
Dot Plot of Sensitivity Analyses of the Effect of Mandate Expansion on Quarterly Maternal Syphilis Case Detection Difference-in-differences results for several sensitivity specifications in addition to the main model. The controls model includes controls for lagged annual state syphilis incidence and compositional characteristics of deliveries (share of births among birthing parents younger than 24 years, with Hispanic ethnicity, non-Hispanic Black race, non-Hispanic White race, who completed high school, who completed college, and who received Medicaid). The exclude any provider letter model excludes any control states that issued provider letters or health alerts. The exclude any screening recommendations model excludes any control states that issued provider letters as well as those that issued sexually transmitted infection screening guidance with recommendations for expanded screening. The balanced panel model starts the panel in 2014, excluding 1 treatment state, Louisiana, due to an insufficient number of pretreatment periods. Whiskers indicate 95% CIs. ^a^*P* < .05. ^b^*P* < .001. ^c^*P* < .01.

ATT estimates across most sensitivity specifications were consistent with the main results (Figure 3). Treatment effects were similar and statistically significant when excluding all control states that had issued any recommendations around expanded screening (17%; 95% CI, 6-30; *P* = .002) and larger when only excluding control states that had issued provider letters or health alerts (25%; 95% CI, 11-39; *P* < .001). A placebo analysis setting the mandate introduction 1 year earlier estimated no significant effects (eFigure 1 in Supplement 1).

**Figure 4.**
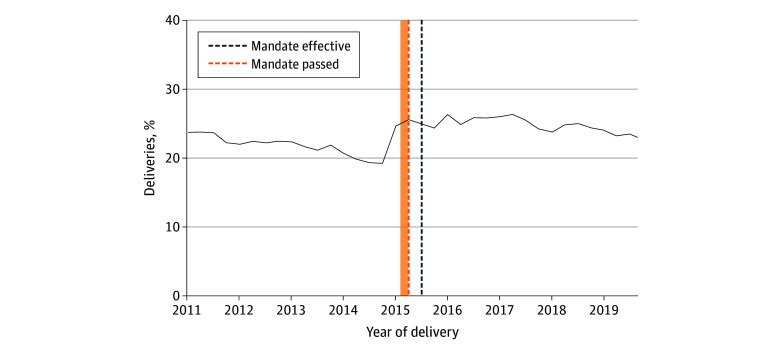
Line Graph of Quarterly Trends in Syphilis Testing at Delivery in Georgia From Inpatient Discharge Records, 2011 to 2019 Trends are plotted for share of all deliveries receiving syphilis screening as reported using *Current Procedural Terminology* codes from the Healthcare Cost Utilization Project State Inpatient Database for Georgia from 2011 to 2019 (eTable 3 in Supplement 1). The shaded area denotes when the bill was introduced into the legislature from the earliest observed date.^30^

Figure 4 presents complementary analyses of temporal trends in screening at delivery in 1 mandate expansion state, Georgia.^30^ Between 2011 and 2019, 1 127 227 deliveries occurred, and of these, 264 126 (23%) were screened for syphilis. Compared with the premandate period (2014 quarter 1 to 2015 quarter 2), the share of all deliveries being screened for syphilis through 2016 increased by almost 4 percentage points (from 21% [32 319 of 156 500] to 25% [56 549 of 222 860]). These results were only suggestive of the impact of the mandate, as much of the change in screening occurred in the quarter prior to when the mandate was enacted.

## Discussion

Despite being preventable with timely testing and treatment, congenital syphilis rates have surged over the last decade, and gaps in screening coverage remain. As of 2025, more than 20 states have mandated expanded prenatal syphilis screening at multiple points in pregnancy and to broader populations. Prior evidence is scant on whether expanded screening improves maternal syphilis case detection and whether mandates are an effective vehicle through which to do so. In national birth certificate data, prenatal screening mandates were associated with a statistically and clinically significant increase in maternal syphilis case detection in the first 2 quarters after they were enacted and in the first quarter after enactment when controlling for covariates. This initial increase in case detection faded within the first year, though remained positive in each of the postmandate quarters assessed. This increase could reflect changes in screening coverage—both changes in the overall share of individuals offered screening and the risk among those offered—as well as changes in underlying syphilis incidence.

Evidence from Georgia suggests that screening at delivery may have responded to a high-risk mandate. Prior descriptive studies indicate that screening coverage may respond to changes in clinical guidelines and state mandates but often remains below universal recommendations. For example, prenatal hepatitis C screening increased 10 percentage points following a change in Centers for Disease Control and US Preventive Services Task Force recommendations, whereas state mandates for universal HIV screening yielded more mixed results.^34,35^

The fade-out of case detection within the first year may reflect declining adherence to mandates among expansion states. Adherence to long-standing prenatal syphilis screening mandates is known to be low, and mandates alone are unlikely to sustain higher coverage without complementary policies that facilitate and monitor clinician adherence to offer screening and reduce barriers to patient uptake.^10,12^ Although several states stipulate penalties for noncompliance, systematic monitoring is rare. Efforts are underway to develop a Healthcare Effectiveness Data and Information Set performance monitoring measure that could help payers and administrators track compliance.^36^ Further, the impact of state-level mandates may be dampened if patients face barriers to uptake, such as insurance cost-sharing or lack of coverage. Qualitative research has documented clinician hesitancy to offer expanded testing when Medicaid reimburses syphilis screening, but private insurance may have more limited reimbursement or impose cost-sharing.^6,37^

Rising case detection among control states—either due to increasing transmission or screening coverage—may also attenuate treatment effects over time. Several control states issued health alerts and provider letters recommending expanded screening.^38,39,40,41^ Excluding these states in sensitivity analyses increased the mean treatment effect up to 5 percentage points. In addition, some mandate expansion states may have issued revised screening recommendations prior to mandates being enacted, potentially dampening estimated effects.^42,43^

Mandates should increase case detection if they induce more screening among higher-risk individuals. However, high-risk screening may be limited if screening is not successfully targeted based on true risk. Existing evidence has found the likelihood of third-trimester screening to be higher among non-Hispanic Black individuals and individuals with public insurance.^10,12^ More evidence is needed on the relative effectiveness of universal vs risk-based screening at reducing potential biases in screening coverage. As syphilis risk becomes more generalized, relying on risk factor–based screening may result in missed opportunities for detection and treatment.^44^

### Limitations

This study has several limitations. First, we could only observe syphilis diagnoses during pregnancy and delivery. We could not observe who was tested, when testing occurred, or test results, so we could not assess which mechanisms drove changes in case detection. Reporting of syphilis infection on the birth certificate is likely measured with error; infections identified earlier in pregnancy may be missed if prenatal records are not accessible. Further, we could not differentiate new infections from individuals with persistent seropositivity after prior treatment. Second, we could not separate the effects of expanded screening itself from the impact of implementing screening through legal mandates. Third, we could not distinguish the effects of repeat screening at the third trimester vs delivery or of universal vs high-risk screening. Finally, we did not assess whether near-term increases in maternal case detection improved downstream maternal and infant outcomes, such as timely treatment initiation or stillbirth prevention.

## Conclusions

Expanded prenatal syphilis screening mandates may improve maternal case detection in the short-term. The initial increase in case detection seen in this study faded within the first year, though remained positive in each of the 4 postmandate quarters assessed. Additional interventions, such as those that monitor and facilitate clinician adherence to offer screening as well as those that support treatment retention and follow-up, are likely needed for sustained impact.

## Notes

Supplement 1. **eMethods.** Model Specifications **eTable 1.** Policy Details for All States That Expanded Mandates Between 2012 and 2022 **eTable 2.** Treatment Group Assignment Used for Analysis **eTable 3.** Procedure and Diagnosis Codes Used to Identify Syphilis Screening and Diagnosis at Delivery **eTable 4.** ATT Estimates for Each Mandate Expansion State **eFigure.** Placebo Test of the Effect of Mandate Expansion on Quarterly Syphilis Case Detection

Supplement 2. Data Sharing Statement
