# Supplementary material for: Prenatal Syphilis Screening Mandates and Maternal Syphilis Case Detection
Source: JAMA Health Forum. 2026 Mar 20;7(3):e260123. doi: 10.1001/jamahealthforum.2026.0123 (PMC13005160; doi:10.1001/jamahealthforum.2026.0123)
Supplement: Supplement 1. — eMethods. Model Specifications eTable 1. Policy Details for All States That Expanded Mandates Between 2012 and 2022 eTable 2. Treatment Group Assignment Used for Analysis eTable 3. Procedure and Diagnosis Codes Used to Identify Syphilis Screening and Diagnosis at Delivery eTable 4. ATT Estimates for Each Mandate Expansion State eFigure. Placebo Test of the Effect of Mandate Expansion on Quarterly Syphilis Case Detection [file jamahealthforum-e260123-s001.pdf]

Supplemental Online Content

Baum SE, Agha L, Menzies NA, Cohen J. Prenatal syphilis screening mandates and maternal syphilis case detection. *JAMA Health Forum.* 2026;7(3):e260123. doi:10.1001/jamahealthforum.2026.0123

eMethods. Model Specifications

eTable 1. Policy Details for All States That Expanded Mandates Between 2012 and 2022

eTable 2. Treatment Group Assignment Used for Analysis

eTable 3. Procedure and Diagnosis Codes Used to Identify Syphilis Screening and Diagnosis at Delivery

eTable 4. ATT Estimates for Each Mandate Expansion State

eFigure. Placebo Test of the Effect of Mandate Expansion on Quarterly Syphilis Case Detection

This supplementary material has been provided by the authors to give readers additional information about their work.

© 2026 Baum SE et al. *JAMA Health Forum*.

eMethods. Model Specifications

Difference-in-differences design:
An event study model of changes in syphilis case detection was estimated using the following specification:

$$log\ (Y_{st})\ = \alpha + \sum_{t=\,-\tau}^{-2} \beta_t \times\ D_{st} + \sum_{0}^{t=\,\tau} \beta_t \times\ D_{st} +\ \gamma_s +\ \lambda_t +\ \varepsilon_{st} +\ log\ (n_{st})$$

where $Y_{st}$was the number of syphilis cases detected and $n_{st}$was an offset term for total number of live births in state *s* in quarter *t*. D_{st} was a binary indicator equal to 1 for mandate expansion states and 0 for control. τ represented time-to-event dummies for each quarter pre- or post- a mandate being enacted (with $\tau\ =\ -1$ omitted as the reference period). γ_s captured state-level fixed effects, λ_t captures time fixed effects, and ε_{st} is the error term. Quarterly count syphilis cases were modeled using a Poisson regression.

Difference-in-differences estimates of the change in syphilis case detection per state-quarter was modeled using a two-way fixed effects Poisson model for 4 quarters after the mandate was enacted. We modeled syphilis case detection in state *s* in quarter *t* as:

$$log\ (Y_{st})\ = \alpha +\ \gamma_s +\ \lambda_t +\ \delta * D_{st} +\ \varepsilon_{st} + log\ (n_{st})$$

where $Y_{st}$was the number of syphilis cases detected and $n_{st}$was an offset term for total number of live births in state *s* in quarter *t*. D_{st} was a binary indicator equal to 1 for quarters where mandates had been enacted in treated states and 0 before the start of the policy, including for all control states. δ is the average treatment on the treated. γ_s captured state-level fixed effects, λ_t captured time fixed effects, and ε_{st} was the error term.

Calculating the average marginal effect following Feng and Bilinski (2025):
For each mandate expansion state, we subtracted the ATT from their observed incidence on the log scale at each quarter in the year after the mandate was enacted and then exponentiated to predict counterfactual incidence if the state would have remained untreated. We calculated the difference between the observed and fitted counterfactual incidences. We averaged over all treated units to get the average marginal effect per quarter, or additional cases detected per 100,000 live births.

© 2026 Baum SE et al. *JAMA Health Forum*.

eTable 1. Policy Details for All States That Expanded Mandates Between 2012 and 2022

State	Policy			Passage Date	Effective Date
	First	Third	Delivery		
Arkansas	U	U		10/2014	12/2014
Louisiana	U	U	HR	06/2014	06/2014
Georgia	U	U	HR	05/2015	07/2015
Arizona	U	U	U	09/2017	01/2018
Michigan	U	U	HR	12/2018	03/2019
California	U	U	U	10/2021	01/2022

Table reports policy details for all states that expanded mandates between 2012-2022, regardless of whether or not they were included in analysis. Arkansas was not included in this analysis because of an insufficient number of pre-periods observed prior to the policy change. California was excluded due to insufficient number of post-treatment observations. Policy details are based on policy descriptions outlined by the CDC's Division of STD Prevention.[14] Under the Policy column, universal (U) indicates that policy stipulates that all pregnant individuals are to be screened. High Risk (HR) definition varied by state, but often included individuals with no evidence of a prior syphilis test result, no prior screening, or indicators of risk for infection.

© 2026 Baum SE et al. *JAMA Health Forum*.

eTable 2. Treatment Group Assignment Used for Analysis

Treatment Group	States
**Mandate Expansion States** (n = 4)	GA, LA, AZ, MI
**Control** (n = 29)	AK, CO, HI, IA, ID, KS, KY, MA, ME, MN, MS, MT, ND, NE, NH, NM, OH, OK, OR, RI, SC, SD, UT, VA, VT, WA, WI, WV, WY
**Always Treated** (n = 18)	**Only third trimester:** AR, CT, DC, DE, IL, IN, PA, TN<br>**Only delivery:** NJ, NY<br>**Both third trimester and delivery:** AL, CA, FL, NC, NV, MD, MO, TX

Allocation of 50 states and D.C. to each treatment group. "Mandate Expansion" states refer to states that enacted an expanded prenatal screening mandate at the third trimester and delivery between 2012-2022. "Control" states refer to states that had not enacted an expanded mandate during this period. This includes states that had only a mandate in the first-trimester as well as 7 states (HI, IA, ME, MN, MS, NH, ND) that had not enacted any mandate prior to 2023. "Always treated" states include any state that had already enacted an expanded mandate prior to 2012 and were not included in this analysis. Arkansas and California enacted mandates between 2012-2022, but were considered "always treated".

© 2026 Baum SE et al. *JAMA Health Forum*.

eTable 3. Procedure and Diagnosis Codes Used to Identify Syphilis Screening and Diagnosis at Delivery

**Type**	
**Current Procedural Terminology (CPT)**	86780, 86781, 86592, 86593, 87285, 87164, 87166, 80055, 80081 (also includes HIV)
**International Classification of Diseases (9 and 10)**	090, 091, 092, 093, 094, 095, 096, 097, 6470, A50, A51, A52, A53, O980, O9811, Z11.3, Z11.59

© 2026 Baum SE et al. *JAMA Health Forum*.

eTable 4. ATT Estimates for Each Mandate Expansion State

	ATT	
	(1)	(2)
Arizona	59.1***<br>(95% CI: 31.3, 92.8)	47.0***<br>(95% CI: 19.6, 80.8)
Georgia	-2.5<br>(95% CI: -20.1, 19.0)	-12.2<br>(95% CI: -26.6, 5.1)
Louisiana	14.0<br>(95% CI: -8.0, 41.1)	5.5<br>(95% CI: -12.7, 27.5)
Michigan	15.8***<br>(95% CI: 6.6, 25.8)	12.2<br>(95% CI: -0.7, 26.9)
Quarter FE	X	X
State FE	X	X
State Controls		X

ATT estimates from a staggered two-way fixed effects specification estimated separately for each mandate expansion state over the first four quarters following when the mandate was enacted. Column 2 controls for lagged syphilis incidence in the population and time-varying demographic controls (e.g. share of births: younger than 24 years old, Hispanic, non-Hispanic White, non-Hispanic Black, completed high school, completed college, received Medicaid). Standard errors are clustered at the state-level with associated 95% confidence intervals. Note: *** $p \leq 0.001$, ** $p \leq 0.01$, * $p \leq 0.05$.

© 2026 Baum SE et al. *JAMA Health Forum*.

eFigure. Placebo Test of the Effect of Mandate Expansion on Quarterly Syphilis Case Detection

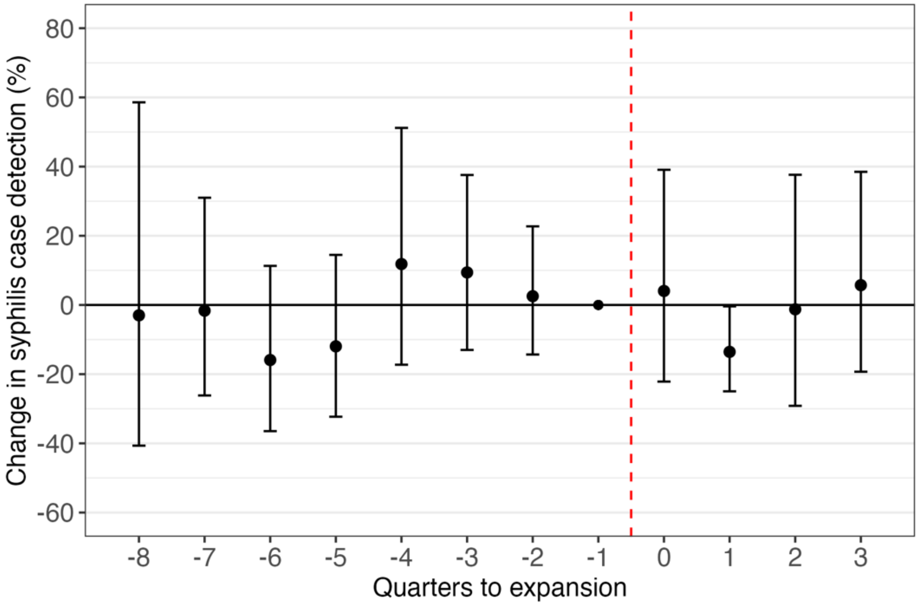

Event study results from an staggered two way fixed effects using a placebo date of when each state enacted their mandate. Specification includes state and quarter fixed effects. Standard errors are clustered at the state-level with associated 95% confidence intervals. Results are presented quarterly relative to the quarter preceding when each state legislature passed their screening mandate.

© 2026 Baum SE et al. *JAMA Health Forum*.
