# Supplementary material for: Prenatal Syphilis Screening Mandates and Maternal Syphilis Case Detection
Source: JAMA Health Forum. 2026 Mar 20;7(3):e260123. doi: 10.1001/jamahealthforum.2026.0123 (PMC13005160; doi:10.1001/jamahealthforum.2026.0123)
Supplement: Supplement 2. — Data Sharing Statement [file jamahealthforum-e260123-s002.pdf]

Data Sharing Statement

Baum. Prenatal Syphilis Screening Mandates and Maternal Syphilis Case Detection. *JAMA Health Forum*. Published March 20, 2026. doi:10.1001/jamahealthforum.2026.0123

Data
Data available: No
